# Audiologic evaluation in patients with acquired hypothyroidism

**DOI:** 10.1590/S1808-86942010000400012

**Published:** 2015-10-19

**Authors:** Karlos Thiago Pinheiro dos Santos, Norimar Hernandes Dias, Gláucia Maria Ferreira da Silva Mazeto, Lidia Raquel de Carvalho, Renan Luis Lapate, Regina Helena Garcia Martins

**Affiliations:** a Speech and Hearing therapist. Graduate student - General Basis of Surgery - Medical School - UNESP - Botucatu; b ENT physician - Otorhinolaryngology Program. PhD in Surgery - Medical School - Unesp- Botucatu; c PhD. Professor of Endocrinology - Medical School of Botucatu; d PhD. Professor - Department of Biostatstcs - Insttute of Biosciences - Unesp-Botucatu; e Medical Student -Unesp- Botucatu; f Associate Professor - Medical School of Botucatu - Unesp. Head of the Phoniatry and Voice Ward

**Keywords:** audiometry, hypothyroidism, audiometry, hearing loss

## Abstract

Hearing loss in hypothyroidism has been reported by many authors but its pathophysiology is unclear.

**Aims:**

to study the audiological evaluation of patients with acquired hypothyroidism.

**Materials and Methods:**

two groups were included: a hypothyroidism group (HG, n-30), and a control group (CG, n-30). Parameters studied: gender, time of hypothyroidism, comorbidities, cochleovestibular symptoms, biochemistry and hormonal exams (TSH, T4), tonal audiometry, TOAEs and BERA.

**Results:**

all participants were women, 70% of the HG had Hashimoto thyroiditis, 60% of the HG had had the diagnostic of the hypothyroidism for at least five years. Depression and hypertension were frequent in HG. All HG patients had altered TSH values and 50% had diminished T4 values. Sensorineural hearing loss was detected in 22 ears from the HG and in seven from the CG. BERA was normal in the CG and altered in 10 ears from the HG, showing L-V increase. TOAEs were absent in 12 ears from the HG and in four from the CG.

**Conclusions:**

HG patients had more cochleovestibular symptoms, higher audiometric thresholds, increase in L-V in the BERA and absence or reduction in TOAEs amplitudes. Such alterations were not associated with THS and free T4 levels.

## INTRODUCTION

One of the most important dysfunctions of the thyroid gland is hypothyroidism (congenital or acquired) in which the production or function of thyroid hormones is impaired, resulting in generalized reduction of the metabolism of all the systems. Hypothyroidism affects 2% of adult women and only 0.2% of men^1^, ^2^, ^3^.

Auditory acuity reduction has been associated with thyroid gland dysfunction and has been described by numerous authors. In 1974, Ritter stressed that hearing loss can be the most common otorhinolaryngological manifestation of congenital and acquired hypothyroidism, and auditory symptoms may happen alone or in association with vertigo and tinnitus^4^. The real incidence of hearing loss in patients with hypothyroidism is still uncertain, and it may affect 25% of the patients with acquired hypothyroidism and 35-50% of the patients with congenital hypothyroidism^5^^,^^6^. The pathophysiological mechanisms of hearing loss in hypothyroidism are not totally unveiled. It is known that in this hormonal disorder there is a reduction in cell energy production, compromising the microcirculation and, consequently, oxygenation and the metabolism of the involved organs. Inner ear structures are also affected, such as the stria vascularis and the Organ of Corti^7^. Thyroid hormones control protein synthesis, the production of myelin and enzymes and the level of lipids in the central nervous system. Moreover, T4 can act as a neurotransmitter. Thus, it is believed that under hypothyroidism, hearing impairment can originate in the cochlea, in the central auditory pathways and/or in the retrocochlear region^8^.

Experimental studies have tried to pinpoint the auditory pathway sites affected by hypothyroidism. Saito et al.^9^, in an experimental study carried out in dogs, found a reduction in the number of neuronal cells in the cerebral cortex and structural changes in the membranous labyrinth and in the petrous portion of the temporal bone. Thus, similarly to what happens in diabetes mellitus, the hypothyroidism-related hearing impairment seems to involve numerous structures^10^, ^11^, ^12^.

Tonal threshold audiometry, Immittance tests and electrophysiological methods such as brainstem auditory evoked potentials (BAEP) and otoacoustic emissions (EAE) have been used to assess patients with hypothyroidism. The results from these auditory evaluations, both clinical and experimental, are not homogeneous. Studies from Ben-Tovim et al.^5^, in rats, and those from Rubinstein et al.^13^, in swine, both with experimentally induced hypothyroidism, show changes in the amplitude and latency of electrophysiological auditory potentials, being reversible after treatment with thyroid replacement hormones. According to Ben-Tovim et al.^5^, the changes seen in the BAEP are directly associated to the serum levels of the free T4. Himelfarb et al.^14^ found a significant drop in the audiometric thresholds and BAEP changes in patients with thyroid gland dysfunction and highlighted the statistically significant relation between hearing loss and low free T4. Similarly to that, Anand et al.^15^ detected reduction in the audiometric thresholds and BAEP changes in 16 patients (from a total of 20 patients) with hypothyroidism without treatment. After the hormonal replacement treatment for a period of 3.7 months, the authors reported a marked improvement in the audiometric threshold; however, the BAEP results maintained altered, there was also a reduction on the absolute latency amplitudes on waves I, III and V and increase in the absolute latency in wave V and interpeaks LI-III and L I-V.

Di Lorenzo et al.^8^, analyzed the auditory evoked potentials from patients with hypothyroidism, before and after the hormonal replacement therapy and observed that 25% of the patients with hypothyroidism with BAEP changes, represented by the interauricular difference above 0.2 ms, also had increase in wave V absolute latency and that of interpeaks LIII-V and LI-V. These changes were maintained even after 6 to 12 months of hormonal replacement therapy, challenging the reversibility of the hearing impairment in these patients.

In subclinical hypothyroidism, results are also controversial. Ozata et al.^12^ did not observe BAEP changes in 20 of their patients. On the other hand, Figueiredo et al.^16^ assessed 23 patients with subclinical disease and found marked differences in the absolute latencies of waves III and V, and interpeaks LI-III, LIII-V and LI-V.

The otoacoustic emissions study is a fast and reliable electrophysiological method which has proven useful in the investigation of cochlear diseases, as well as in assessing hearing in patients with metabolic disorders. Khechinaschvili et al.^17^, assessing the hearing acuity of 50 patients with hypothyroidism, found that the otoacoustic emissions were absent in some patients who had normal audiometric thresholds. They also found changes in the BAEP of 30% of the patients, suggesting that the auditory involvement in patients with hypothyroidism can be multisectorial.

Considering the above, we notice that the results from the audiological evaluation of patients with hypothyroidism are conflicting, making it necessary to broaden the studies in this line of research. Thus, the goal of the present study was to assess the auditory acuity of patients with acquired hypothyroidism, using tonal threshold audiometry, brainstem auditory evoked potentials and transient otoacoustic emissions.

## MATERIALS AND METHODS

This study was approved by the Ethics in Research Committee of the University where the study was carried out (protocol # 2023/2006). We setup two study groups: a group with hypothyroidism (GH), made up of 30 patients with acquired hypothyroidism, confirmed by clinical and laboratory exams, and the control group (GC), made up of 30 volunteers without thyroid gland disease, belonging to the corresponding age range, submitted to the same evaluation sequence used in the study group. The following exclusion criteria were used: be older than 60 years, having been submitted to prior ear surgery, having an altered otoscopic exam, work in a noisy environment or having an audiometric exam with a result matching that of noise-induced hearing loss, having a conductive hearing loss or type B or C tympanometric curve, report the use of ototoxic medication, report prior history of hereditary hearing loss, report hearing loss since childhood or having a genetic syndrome.

The clinical parameters investigated were: age, hypothyroidism diagnosis time, auditory and/or vestibular symptoms, biochemical test results (fasting glucose, triglycerides, total cholesterol) and hormonal exams (TSH, free T4). These values were deemed normal for the parameters: free T4 and TSH, between 0.8-1.9 ng / dL and 0.4-4.0 mUI / mL, respectively; fasting glucose between 70 and 99 mg / dL; total cholesterol below 200 mg / dL; triglycerides below 150 mg / dL.

For the auditory evaluations we used the following tests: tonal threshold audiometry (A321 Amplaid audiometer, Italy), Immittance measures (775 Amplaid device, Italy), Transient Otoacoustic Emissions test - TOAE (ILO 288 Echoport, Otodynamic Ltd., England) and Brainstem Auditory Evoked Potential - BAEP (Nihon Koden - Neuropack MEB 7102 k device, Japan).

The audiometric results were classified as to type and degree. As far as type is concerned, the losses were classified in: conductive (airway thresholds above 25 dB and the normal bone conduction threshold, with an airbone gap); mixed (air and bone conduction thresholds above 25 dB, with air-bone gap) and sensorineural (air and bone conduction thresholds above 25 dB, without air-bone gap). The patients with conductive or mixed hearing loss were taken off the study. Concerning the grade, the hearing losses were classified into: mild (thresholds between 26 and 40 dB); moderate (thresholds between 41 and 70 dB); severe (thresholds between 71 and 90 dB) and profound (thresholds above 91 dB).

We included in the study those individuals with type A tympanometric curve. The criteria used to classify TOAE as present or absent were: signal reproducibility above 50% and signal amplitude of 3 dB SPL response above the noise spectrum in one or more frequencies. In the study of the brainstem auditory evoked potentials (BAEP) we used the monaural stimulus through filtered clicks (between 300 and 4.000 Hz), lasting for 0.1 milliseconds, frequency of 17 clicks per second and thin polarity.

The stimulus intensity was of 90 DbSPL, and 2,000 clicks were used, within an analysis time of 10ms, keeping the electrodes impedance below 5 Kohms.

Our statistical methods to study the associations between the variables and comparison of proportions between the groups were the chi-square test, the Fisher's exact test and the Goodman test. In comparing the attributes with normal distribution, we used the t Student test, considering 5% as level of significance.

## RESULTS

All participants in this study were women and 70% of them had hypothyroidism secondary to Hashimoto's thyroiditis. In both groups, there was a predominance of the age range between 31 and 50 years over the remaining age ranges (p<0.05). Most of the patients with hypothyroidism (60%) had known of the diagnosis for less than five years. Subclinical hypothyroidism, in which TSH values were altered, keeping normal values of free T4, was seen in 50% of the patients with hypothyroidism. Among other etiological diagnosis of hypothyroidism, we stress idiopathic, total or partial thyroidectomy.

Most of the participants with hypothyroidism did not have comorbidities (53.34%; p<0.05). Depression was diagnosed in 26.66% of the remaining patients, and hypertension in 13.34

Cochleovestibular symptoms were reported by participants from both groups, they were, however, more frequent in those patients with hypothyroidism (p<0.05), highlighting hearing loss (13.33%), tinnitus (16.67%) and vertigo (3.33%). We highlight that 26.67% of these patients had the three associated symptoms (Table 1). Most of the patients from the control group did not report cochleovestibular symptoms (73.33%).

**Table 1:** List of the cochleovestibular symptoms reported by the participants from both study groups.

Symptoms	Groups			
	Control N %		Hypothyroidism N %	
No symptoms	22	73.33a A	7	23.33b A
Tinnitus	5	16.67a B	5	16.67a A
Hearing loss	1	3.33a B	4	13.33a A
Hearing loss and tinnitus	2	6.67a B	5	16.67a A
Hearing loss, tinnitus and vertigo	0	0.00b B	8	26.67a A
Vertigo	0	0.00a B	1	3.33a A
TOTAL	30	100.00	30	100.00

χ^2^ = 19.8; p-0.001

In the comparative analysis of the biochemical exams (fasting glucose, triglycerides and total cholesterol) from the participants of both groups, we did not notice statistically significant differences (p> 0.05). Diabetes mellitus was not seen in any of the participants. Table 2, Table 3, Table 4. High TSH levels were seen only among participants with hypothyroidism, and 50% of them also had reduced levels of free T4.

**Table 2:** Fasting glucose serum levels of the participants from both study groups (mg/dL).

Fasting glucose (mg/dL)	Groups			
	Control N %		Hypothyroidism N %	
< 100	27	90.00a A	18	60.00b A
de 100 a 125	3	10.00b B	12	40.00a A
> 125	0	0.00a B	0	0.00a B
TOTAL	30	100.00	30	100.00

χ^2^ = 7.200; p – 0.007

**Table 3:** Distribution of the patients from both groups in relation to total cholesterol serum levels (mg/dL).

Total cholesterol (mg/dL)	Groups			
	Control N %		Hypothyroidism N %	
< 200	19	63.34	12	40.00
de 200 a 239	8	26.66	11	36.66
> 239	3	10.00	7	23.34
TOTAL	30	100.00	30	100.00

χ^2^ = 3.654; p – 0.301

**Table 4:** Distribution of the participants from both groups in relation to their serum levels of triglycerides (mg/dL).

Triglicérides (mg/dL)	Groups			
	Control N %		Hypothyroidism N %	
≤150	25	83.34	20	66.67
≥151	5	16.66	10	33.33
TOTAL	30	100.00	30	100.00

χ^2^ = 2.222; p – 0.136

**Figure 1:**
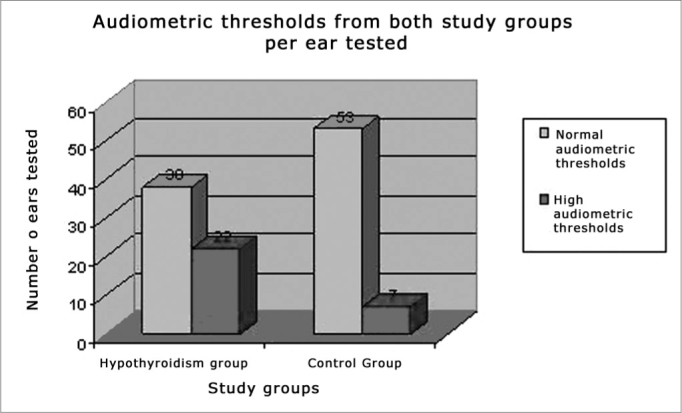
Audiometric thresholds in both study groups per ear tested.

High audiometric thresholds were recorded from 22 ears (36.67%) of the patients with hypothyroidism and in only seven ears (11.67%) from patients of the control group (p<0.05), there was a predominance of mild/moderate sensorineural hearing loss in both groups (Figure 1). There was no significant association between the changed audiometric thresholds and the serum levels of glucose, triglycerides, total cholesterol, TSH and free T4 (p>0.05).

The study of the brainstem auditory evoked potentials (BAEP) was changed in 10 ears from patients with hypothyroidism and in none of the participants from the control group (p<0.05). The mean values from the L-V absolute latencies proved statistically higher in the group with hypothyroidism (p <0.05) (Table 5). The other BAEP parameters have similar behavior in both groups.

**Table 5:** Absolute latencies of waves PI, PIII and PV and of interpeaks LI-III, LIII-V and LI-V (ms) in both study groups.

Mean and standard deviation (DP) of the absolute latencies and interpeaks (ms)	Control Mean SD		Hypothyroidism Mean SD	
L-I	1.58	±0.12	1.61	±0.10
L-III	3.67	±0.13	3.70	±0.19
L-V*	5.57	±0.20	5.65	±0.20
L I-III	2.08	±0.13	2.08	±0.19
L III-V	1.91	±0.17	1.94	±0.16
L I-V	3.99	±0.20	4.02	±0.21

* p<0.05 (t Student Test).

The transient evoked otoacoustic emissions were not present in a higher number of patients with hypothyroidism (20%) when compared to the control group (6.67%) (p<0.05). The transient otoacoustic emission (TOAE) amplitudes were lower in the group of patients with hypothyroidism (p <0.05). In both groups, the values from the emissions amplitudes were lower in the higher frequencies (Table 6).

**Table 6:** Mean values and standard deviation of the of the transient otoacoustic emissions (TOAE) in each frequency from both groups.

	Mean values of the transient otoacoustic emissions (dB)			
Frequency bands (KHz)	Control		Hypothyroidism	
	Mean	SD	Mean	SD
1KHz	12.08	±4.17	8.20	±3.12
1.5KHz	11.33	±3.87	7.06	±2.67
2KHz	9.24	±5.45	5.80	±2.13
3KHz	7.19	±4.04	5.00	±1.76
4KHz	5.83	±2.57	4.13	±0.99

p<0.05 (“t” Student test)

## DISCUSSION

In the present study, among patients with acquired hypothyroidism, Hashimoto's thyroiditis was the main cause (70%), and such result was already expected because it was the main cause of auto-immune hypothyroidism, in which the glandular parenchyma is invaded by a lymphoplasmocytic infiltrate, producing antibodies against the gland^18^. We stress that 50% of the patients had been diagnosed with subclinical hypothyroidism, characterized by normal serum concentrations of free T4 and high levels of TSH. In subclinical hypothyroidism, Hashimoto's thyroiditis is also the main cause, corresponding to 50-80% of the cases^1^, ^2^, ^12^, ^18^, ^19^.

In relation to the TSH and free T4 values, we noticed that all the patients with hypothyroidism had changed serum levels of TSH. One must consider the fact that all these patients were periodically followed up in endocrinology wards, they were under hormonal replacement therapy, and even then they had changed levels of TSH, with or without changes in the levels of free T4.

The analysis of cochleovestibular symptoms showed that approximately 73% of the volunteers in the control group did not have symptoms in this area, nonetheless, some of them reported tinnitus alone, similarly to those patients with hypothyroidism. Tinnitus is a frequent complaint in the general population, affecting from 35 to 40% of adults, and it has multiple causes. Associated symptoms of tinnitus, vertigo and hearing loss were reported by 26.67% of the patients with hypothyroidism, indicating a possible joint involvement of the cochlear and vestibular systems. Cochleovestibular symptoms associated with metabolic disorders are relatively frequent, being chronic and permanent^20^, ^21^, ^22^, ^23^.

High audiometric thresholds were recorded in a larger group of patients with hypothyroidism (22 ears) when compared to the patients in the control group, and most of them had mild to moderate and bilateral sensorineural hearing loss. The pathophysiology of the hearing involvement in metabolic disorders has been studied by many researchers. The authors consider that many auditory pathway sites are involved in this process, and endocochlear, retrocochlear and central structures may be involved^11^^,^^17^.

In the present study, the most prevalent co-morbidities were depression (26.66%) and hypertension (13.34%). The greatest prevalence of hypertension among individuals with hypothyroidism was previously reported by Kotsis et al.^24^ who recorded higher levels of systolic arterial pressure in the group of patients with hypothyroidism, when compared to the control group. It is known that hypertension is an important predisposing factor to hearing impairment, since it affects the cochlear duct structures, especially the stria vascularis, which is sensitive to pressure variations.

Other important risk cofactors for hearing loss are changes to the biochemical tests. In the present study, regarding fasting glycemia, we noticed that 40% of the patients with hypothyroidism had moderately high levels of fasting glucose (between 100 and 125 mg / dL), nonetheless, no patient had been diagnosed with diabetes. It is very likely that these mildly changed glucose levels did not cause hearing impairment. Nonetheless, we stress the need for a more in-depth study on the metabolism of carbohydrates in order to better interpret the results, such as the glucose tolerance test and the insulinemic curve, since the literature is full of publications showing the high incidence of cochleovestibular symptoms and hearing impairment in diabetic patients^22^^,^^23^.

Lipid metabolism disorders may also cause cochleovestibular symptoms and compromise inner ear structures. In a clinical study, Karlidag et al.^25^ carried out audiometry tests in 274 patients with dyslipidemias and in 60 healthy individuals, observing higher audiometric thresholds in patients with lipid disorders. By analyzing the results from this study, we notice that the triglyceride and total cholesterol serum levels were discretely higher in both groups, and were probably not associated with changed audiologic tests.

As we analyzed the patients with changed audiometric tests, we observed that 75% of them had TSH values between 4 and 30 mUI / mL and in only a handful of cases the TSH serum levels were very high. Changed audiometric tests were also seen in patients with proper free T4 levels. Thus, the audiometric thresholds do not seem to be associated with the serum levels of these hormones.

Altered BAEP values were seen in 10 ears from patients with hypothyroidism, with higher absolute latencies of waves I, III and V when compared to the control group; however, only the L-V values were statistically different (p<0.05). The diagnosis of retrocochlear lesions in hypothyroidism had been already stressed by Anand et al.^15^ in 80% of their patients with hormonal disorders, and such index was much higher than the one seen in the present study. Important BAEP changes were also seen by Figueiredo et al.^16^ in patients with subclinical hypothyroidism, stressing the increase in the absolute latency of waves I and V, as well as that of interpeaks LI-III, LIII-V and LI-V.

Transient otoacoustic emissions were absent in a larger number of people with hypothyroidism. In interpreting the results from otoacoustic emissions, it is important to assess not only the presence or absence of the curve, but also the response amplitude. They were systematically reduced in all the frequencies analyzed in the group of patients with hypothyroidism, when compared to those in the control group.

## CONCLUSIONS

Cochleovestibular symptoms were more frequent in patients with hypothyroidism, stressing tinnitus, hearing loss and vertigo. Patients with hypothyroidism have a higher number of changes in audiometric evaluations and in electrophysiological tests (BAEO and TOAE). The hearing loss was sensorineural, from mild to moderate in intensity and bilateral. In the BAEP, the increase in the absolute latency of L-V was the most significant change. Patients with hypothyroidism had no otoacoustic emission or had them with lower amplitudes more frequently than their control group counterparts. Audiological changes were not associated with TSH or free T4 serum levels.
